# What role do miRNAs play in the injured heart of the infertile female?

**DOI:** 10.1016/j.bbrep.2025.102382

**Published:** 2025-11-25

**Authors:** Amir Hossein Mohammadi, Parvin Yavari

**Affiliations:** a Research Center for Bochemistry and Nutrition in Metabolic Diseases, Institute for Basic Sciences, Kashan University of Medical Sciences, Kashan, Iran; b Student Research Committee, Kashan University of Medical Sciences, Kashan, Iran; c Regenerative Medicine Research Center, Isfahan University of Medical Sciences, Isfahan, Iran

**Keywords:** Non-coding RNAs, miRNAs, Exosome, Infertility, Cardiovascular diseases

## Abstract

Due to numerous overlapping risk factors between cardiovascular diseases (CVDs) and infertility, women of reproductive age with underlying CVD may increasingly seek reproductive assistance. miRNAs and exosomal miRNAs are well-known epigenetic modulators that regulate gene expression at the post-transcriptional level. A large body of evidence indicates the fundamental roles of various miRNAs in different aspects of both CVD and infertility. Dysregulation of miRNAs (including those carried by exosomes) could contribute to pathological alterations involved in CVD pathogenesis, potentially leading to female infertility-related disorders In particular, we emphasize how these miRNAs shape inflammatory, angiogenic and remodeling responses in the injured myocardium of infertile women. Herein, we review the roles of miRNAs in the development of CVD and female infertility. We also summarize recent research on the role of miRNAs in angiogenesis, including their targets and mechanisms of action. Understanding these mechanisms may facilitate the development of composite biomarkers and targeted diagnostic/therapeutic strategies for female infertility and cardiovascular disease, with a specific focus on myocardial injury.

## Highlights

• MicroRNAs play dual roles in infertility and cardiovascular disease development.
• Exosomal MicroRNAs are novel mediators of cell communication in reproductive health.
• Dysregulated miR-155 and miR-185 are linked to inflammation and female infertility.
• MiR-21 and miR-451 in exosomes may serve as therapeutic targets for CVD and infertility.
• Combining MicroRNAs with traditional markers improves early diagnosis and prognosis.

## 1 Introduction

Infertility defined as a disease of the reproductive system, affects many couples worldwide [1]. Several lines of evidence suggest that infertility can be an early indicator of future health risks in women. In fact, infertility and cardiovascular diseases (CVDs) share a number of common pathways, often mediated by endocrine and metabolic conditions such as polycystic ovarian syndrome (PCOS), hypothyroidism, metabolic syndrome, and endometriosis [[2], [3], [4], [5]]. According to the World Health Organization, CVDs account for approximately one in three deaths worldwide [6]. Psychological stress may be one mediating factor between infertility and CVD; infertility itself can trigger significant stress [7], which in turn elevates the risk of CVD [8]. Furthermore, shared genetic susceptibilities, epigenetic disturbances, and exposure to endocrine disruptors have been shown to adversely affect both reproductive health and cardiovascular health [9].

Non-coding RNAs have recently emerged as critical regulators of gene expression, with a fundamental capacity to modulate numerous genes [10]. They exert their functions through multiple processes at the post-transcriptional and post-translational levels [11]. Among non-coding RNAs, miRNAs have gained particular attention as mediators of paracrine signaling and regulators of cell–cell communication in both physiological and pathological conditions. miRNAs are short (∼22 nucleotide) non-coding RNAs that typically repress translation or induce degradation of target mRNAs. Notably, infertility and CVD also share strong genetic and epigenetic bases [9], and studies have revealed that miRNAs play critical roles in the initiation and progression of female infertility-related disorders [12]. Likewise, altered miRNA expression is linked to the development and progression of various CVDs, such as cardiac hypertrophy and heart failure. The discovery of circulating extracellular miRNAs has uncovered a novel role for miRNAs as paracrine signaling mediators. Circulating miRNAs are actively transported in the bloodstream by binding to RNA-binding proteins or by encapsulation in microvesicles/exosomes, which protects them from ribonuclease-dependent degradation. Notably, a new form of miRNA-based intercellular communication was recently discovered, wherein vesicle-encapsulated miRNAs mediate signals between endothelial cells and other cardiovascular cells [13,14].

**Fig. 1:**
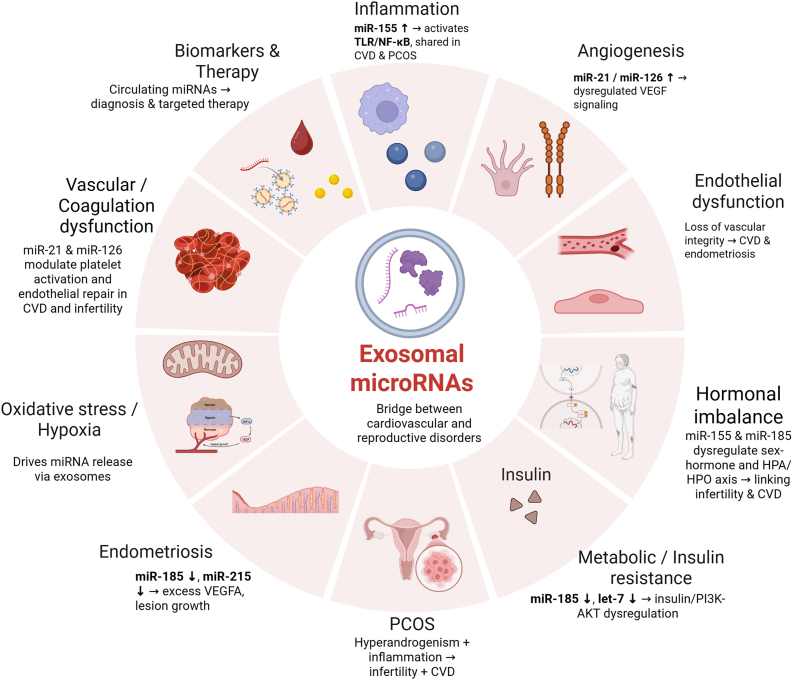
Summary of exosomal microRNAs linking female infertility and cardiovascular disease. Exosomal miRNAs (miR-21, miR-126, miR-155, miR-185, miR-215) mediate shared molecular pathways including inflammation, angiogenesis, endothelial and hormonal dysfunction, metabolic imbalance, and vascular abnormalities, forming a mechanistic bridge between cardiovascular and reproductive disorders and offering translational potential as biomarkers and therapeutic targets.

In this review, we aim to investigate the role of miRNAs, including exosomal miRNAs as a potential bridge between female infertility and CVD (Fig. 1). We first provide an overview of infertility and CVD and examine evidence for their interrelationship. We then describe the biogenesis of miRNAs, followed by a discussion of specific miRNAs and exosomal miRNAs that have been implicated in both infertility and CVD. Finally, we highlight emerging therapeutic strategies targeting these miRNAs. Understanding these connections will help in establishing novel biomarkers and developing innovative diagnostic or therapeutic approaches for women's reproductive and cardiovascular health.

## 2 Infertility

Infertility is often categorized as primary or secondary. Primary infertility refers to couples who have never been able to conceive, whereas secondary infertility refers to those who are unable to conceive after a prior pregnancy (including those who had a miscarriage) [15,16]. Infertility can result from both female and male factors [17]. In females, ovulation disorders and uterine abnormalities are among the most common causes of infertility [18]. In males, common causes include inadequate production of functional sperm (e.g. low count or poor morphology) and reduced sperm motility [19]. Overall, male infertility factors are commonly grouped into defects in spermatogenesis, problems with sperm transport, anti-sperm antibodies, disorders of intercourse (such as impotence), and endocrine disturbances affecting hormonal regulation of reproduction [20].

A variety of gynecological and systemic conditions can lead to female infertility. Cervical factor infertility, for example, occurs when sperm cannot successfully traverse the cervix. Causes include the presence of anti-sperm antibodies in cervical mucus, cervical stenosis (narrowing), inadequate or “hostile” cervical mucus, or chronic cervical infections (often due to sexually transmitted pathogens like *Mycoplasma hominis, Chlamydia trachomatis, Ureaplasma urealyticum, Trichomonas vaginalis,* or *Neisseria gonorrhoeae*) [21,22]. Damage to the fallopian tubes – often from pelvic inflammatory disease or surgeries – can prevent the egg and sperm from meeting, thereby causing infertility [23]. Hormonal imbalances or ovulatory dysfunctions (e.g. due to premature ovarian failure or thyroid disorders) can also lead to infertility. A wide range of medical conditions are associated with female infertility, including endometriosis PCOS, uterine fibroids, pelvic inflammatory disease, and even environmental exposures that disrupt hormonal function [23]. Among the primary causes of female infertility are tubal blockage, ovulation problems, uterine abnormalities, advanced maternal age, and hormonal imbalances; in contrast, the leading single cause of male infertility is poor semen quality (low count or motility and abnormal morphology) [23].

Chronic low-grade inflammation has been implicated in infertility, particularly in men. For instance, certain miRNAs that are upregulated in activated macrophages contribute to inflammatory pathologies and may affect reproductive function. Male subfertility is often associated with low testosterone levels, and a growing body of evidence links low testosterone with metabolic syndrome and low-grade systemic inflammation (LGSI) – both of which are risk factors for CVD. One study found that young subfertile men with low androgen levels also exhibited elevated inflammatory cytokines and chemokines, suggesting a connection between low testosterone, chronic inflammation, and male subfertility. Further data support an important role of inflammation in metabolic and endocrine disorders that might underlie male infertility. In female infertility, aberrant inflammation is exemplified by endometriosis, in which tissue similar to the endometrium grows outside the uterus. miRNAs are involved in the pathogenesis of endometriosis by modulating processes like angiogenesis, cell proliferation, inflammation, and tissue remodeling [24]. Indeed, several miRNAs are upregulated in severe endometriosis in eutopic endometrial tissue and have been proposed as potential non-invasive biomarkers of the disease [25].

## 3 CVD

CVDs are the leading global cause of death. We first outline their global burden, then summarize non-coding RNA involvement in pathogenesis, emphasize miRNAs as regulators/biomarkers, and finally link these mechanisms back to infertility [6]. CVDs which include coronary artery disease, stroke, heart failure, and others are the leading cause of death worldwide [26]. Despite significant advances in primary prevention and treatment, the overall prevalence of CVD has continued to rise in recent years, partly due to aging populations and lifestyle factors. An in-depth understanding of the molecular pathophysiology of CVD is essential for discovering novel biomarkers that enable early and accurate diagnosis and for developing effective preventive strategies.

It is now recognized that while approximately 80 % of the human genome is transcribed, only about 1–2 % of the genome encodes proteins. The remaining transcripts are non-coding RNAs of various classes [27,28]. These include small nuclear RNAs, small nucleolar RNAs, piwi-interacting RNAs, long non-coding RNAs, and the focus of this review – miRNAs. Non-coding RNAs are crucial regulators of gene expression and play significant roles in epigenetic regulation of cellular functions. miRNAs, in particular, are the most well-characterized class of non-coding RNAs in the cardiovascular literature, and they are increasingly understood to be important etiological factors in the development of CVD [29].

Multiple studies have demonstrated fundamental roles for miRNAs in maintaining vascular integrity and homeostasis. miRNAs are recognized as both mediators and potential biomarkers of various vascular diseases. For example, certain miRNAs are required for normal function of endothelial progenitor cells (EPCs) that repair blood vessels; downregulation of these miRNAs can impair EPC function and thus vascular repair. Conversely, EPC-derived microvesicles carrying specific miRNAs have been shown to protect against ischemia–reperfusion injury in animal models by transferring these miRNAs to target cells. miRNAs also regulate angiogenic processes; one mechanism is through modulation of the vascular endothelial growth factor (VEGF) signaling pathway. For instance, miRNA-mediated downregulation of the VEGF receptor 2 on endothelial cells can affect new blood vessel formation. EPC-derived microvesicles were found to influence endothelial cell function and apoptosis via the miRNAs they carry. These findings underscore that miRNAs play diverse and critical roles in cardiovascular biology.

## 4 Association between infertility and CVD

The relationship between female infertility and cardiovascular disease risk is complex and not yet fully established. However, there are plausible biological links and an increasing number of studies investigating this association. Both infertility and CVD are associated with aging and often share underlying conditions. For example, women who experience infertility at older ages frequently have conditions like PCOS or obesity, which make them prone to gestational diabetes and pregnancy-related hypertension – factors that subsequently increase their later risk of CVD (including heart disease and stroke) [22]. PCOS is a major cause of anovulatory infertility and is closely associated with metabolic syndrome, thereby increasing the risk of atherosclerotic cardiovascular disease in affected women. Inferring a direct causal relationship between infertility and CVD, however, is challenging due to the multitude of confounding factors [21]. Additionally, women who are infertile due to early menopause (premature ovarian failure) have been found to be at higher risk of CVD, underlining the prominent role of age and hormonal status in both reproductive capability and cardiovascular health [21,23].

Vitamin D deficiency is another factor that has been linked to both CVD and infertility. Low vitamin D levels can contribute to the development of atherosclerosis, and numerous studies also highlight vitamin D's importance in reproductive health (for ovulatory function, endometrial receptivity, etc.) [21]. Another mechanism that might explain the infertility-CVD connection involves over-activation of the renin–angiotensin–aldosterone system (RAAS) during certain infertility treatments. For instance, ovarian hyperstimulation (as part of some assisted reproductive techniques) directly activates RAAS, leading to interstitial edema, increased vascular permeability, and endothelial dysfunction. These changes, in severe cases (such as ovarian hyperstimulation syndrome), can precipitate cardiovascular complications like heart failure or ischemic stroke [21]. Neuroendocrine dysfunction may also link the two conditions; disturbances in the hypothalamic–pituitary–adrenal axis can alter sex hormone levels, resulting in reproductive dysfunction and features of metabolic syndrome, thereby increasing CVD risk [21]. In summary, there are several pathophysiological pathways (endocrine, metabolic, inflammatory, etc.) that could jointly underlie infertility and cardiovascular risk.

Empirical research on the infertility–CVD relationship has yielded mixed results, with some studies reporting positive associations and others finding no significant link. For example, one study comparing 117 infertile women to 627 fertile women found that the infertile group had a significantly higher likelihood of cardiovascular risk factors and early signs of CVD [24]. However, another study with a larger control group reported no significant difference in cardiovascular outcomes between infertile and fertile women [9]. In contrast, a large cohort study demonstrated that subfertility (difficulty conceiving for an extended period) is a risk factor for later CVD among women who eventually do have a child, even after accounting for traditional cardiovascular risk factors and adverse pregnancy outcomes [25]. In one analysis, women with a history of five or more years of infertility had about a 19 % higher risk of developing CVD compared to women with no infertility, highlighting that prolonged subfertility might be an early warning sign for cardiovascular issues. Intriguingly, another study showed that the risk of ischemic stroke and CVD was actually lower in women who had received fertility drugs compared to untreated infertile women, suggesting a potential protective effect of successful ovulation induction or other unmeasured healthy-user effects in those who undergo treatment [26]. Meanwhile, data on the impact of infertility treatment outcomes on CVD risk are still limited. A recent cohort analysis of women with unexplained infertility who underwent in vitro fertilization (IVF) found that women for whom IVF was unsuccessful had an increased risk of developing CVD during a mean follow-up of 8.4 years, compared to those who eventually had a successful pregnancy. Therefore, a failure to conceive even with treatment or a longer duration of infertility, may be associated with a higher long-term risk of cardiovascular disease [27].

There is also interest in understanding how specific cardiovascular risk factors (like hypertension, diabetes, and dyslipidemia) correlate with infertility. Overall, data are somewhat limited, but emerging studies suggest some notable patterns. For instance, the risk of developing type 2 diabetes appears to be higher in women with a history of infertility (particularly for those with idiopathic infertility, tubal factor infertility, or ovulation disorders) compared to women without fertility issues [28]. In one prospective study, infertility was associated with a greater incidence of diabetes, and this connection was hypothesized to be partly due to underlying hormonal or metabolic imbalances common to both conditions. Similarly, another analysis indicated that among women under 45, those with infertility (especially due to ovarian dysfunction) had higher rates of new-onset diabetes and hypercholesterolemia than their fertile counterparts; in women aged 45 or above, those with infertility had higher rates of hypercholesterolemia and hypertension as well [29]. Furthermore, a cohort study observed that infertile women tend to have higher body mass index and were significantly more likely to be obese than fertile women of the same age, suggesting that adiposity could be a confounding link between infertility and cardiovascular risk [30]. In terms of hypertension (high blood pressure), one study reported an increased risk of hypertension in women whose infertility was attributed to a tubal factor, compared to fertile controls [31]. Another study found that women who eventually gave birth after infertility treatment had a lower incidence of chronic conditions (including dyslipidemia, diabetes, and hypertension) than women who remained childless despite treatment, implying that successful resolution of infertility might mitigate some health risks [32]. Additionally, women who underwent IVF have been noted to have a higher risk of developing hypertension later, compared to women who conceived naturally when matched for age and delivery year [33]. Altogether, these findings support an association between infertility and cardiovascular risk factors (such as metabolic syndrome components), although causal relationships remain to be clarified.

Two specific reproductive disorders PCOS and endometriosis, In large Danish‐registry cohorts, women with PCOS had an adjusted HR = 1.58 (95 % CI 1.21–2.07) for acute myocardial infarction, and an adjusted HR = 1.56 (95 % CI 1.27–1.91) for ischaemic stroke [34]. Similarly, women with Endometriosis exhibited an adjusted HR = 1.15 (95 % CI 1.11–1.20) for major cardiovascular events (composite of MI and stroke) [35]. exemplify the overlap between infertility and CVD risk. Women with PCOS are at increased risk of metabolic disturbances (insulin resistance, dyslipidemia, central obesity), which are established risk factors for CVD [21]. Large studies and meta-analyses have found that women with PCOS have a higher incidence of coronary heart disease and stroke compared to women without PCOS [21]. This elevated cardiovascular risk is thought to be largely mediated by the PCOS commonly features low high-density lipoprotein cholesterol, elevated triglycerides, increased total and low-density lipoprotein cholesterol, chronic low-grade inflammation, hypertension, and insulin resistance [21]. In other words, PCOS appears to be positively associated with CVD incidence, and this can be explained by its strong links to traditional CVD risk factors.

Endometriosis, a condition associated with chronic pelvic inflammation and infertility, has also been linked to higher cardiovascular risk. In a 20-year cohort study, women with endometriosis had a significantly greater risk of myocardial infarction and angina, and they exhibited higher circulating levels of inflammatory markers, compared to women without endometriosis [36]. Moreover, vascular function studies have shown that women with endometriosis have impaired endothelial function. For example, flow-mediated dilation of the brachial artery, a measure of endothelial health was significantly lower in women with endometriosis than in healthy controls of similar age [37,38]. This endothelial dysfunction in endometriosis patients could be due to the pro-inflammatory and pro-angiogenic state associated with the disease. In fact, endometriosis is often accompanied by increased prevalence of hypertension and hyperlipidemia, as well as elevated inflammatory cytokines, which together may contribute to the heightened cardiovascular risk profile in these women. In summary, both PCOS and endometriosis illustrate how reproductive disorders that cause infertility can also confer an increased risk of CVD, likely through shared metabolic and inflammatory pathways.

## 5 Central hypothesis and organizing framework

We organize this review around four mechanistic axes that concurrently operate in female infertility and cardiovascular disease (1) Inflammation-innate immunity (e.g., miR-155/miR-146a), (2) angiogenesis.-endothelial integrity (miR-21/miR-126/miR-185), (3) fibrosis-remodeling (miR-21/miR-17∼92), and (4) metabolic–hormonal signaling (miR-185/miR-34c). Throughout, we highlight how these axes converge on the injured myocardium and on infertility models (PCOS, endometriosis, premature ovarian insufficiency).

## 6 Emerging role of genetic/epigenetic mechanisms and miRNAs

While a number of exosomal miRNAs, including miR-126, the miR-17∼92 cluster, and the miR-200 family have been implicated in either cardiovascular disease or reproductive disorders, the overlap of these miRNAs in the specific axis of infertility-associated myocardial injury is less well characterized in the current literature. For example, miR-126 is well documented in endothelial integrity and angiogenesis in cardiovascular settings [39,40], but female-infertility specific data are scarce [41,42]. Similarly, although the miR-17∼92 cluster is known for its role in cardiac development and remodeling [43,44], its direct study in infertility-heart crosstalk is minimal. Given the scope of this review, we therefore focus on the most robustly studied miRNAs (miR-21, miR-155, miR-146a, miR-34c) as bridging molecules between cardiac injury and female infertility, and highlight other candidates as future directions. miRNAs are embedded within complex genetic and epigenetic regulatory networks, and their dysregulation not only reflects but also actively drives pathophysiological processes. In the context of cardiovascular injury in infertile women, exploring miRNAs through the lens of epigenetic regulation offers both mechanistic insight and translational promise.+8

miR-21 is a prototypical “onco/angiomiR” widely studied in cardiovascular diseases. In cardiac fibroblasts and endothelial cells, miR-21 modulates the PTEN/AKT, TGF-β/SMAD, and Sprouty1/ERK–MAPK pathways, exerting pro-angiogenic and anti-apoptotic functions yet also promoting fibrosis in a chronic setting [45]. Elevated circulating miR-21 levels have been reported in patients with acute myocardial infarction and heart failure, suggesting utility as a non-invasive biomarker of myocardial injury and remodeling [46]. Modulation of miR-21 (via mimic or antagomir) has shown benefit in experimental myocardial infarction and heart failure models. However, its dual role protective in early ischemia, yet fibrotic in late remodeling mandates phase- and cell-specific targeting [47].

miR-155 is a key driver of innate immune activation via TLR/NF-κB signaling, implicated in macrophage infiltration, inflammation, and atherosclerosis. Elevated miR-155 levels have been shown in myocarditis and vascular injury models [48]. Because infertility and cardiovascular disease share inflammatory pathways, circulating/exosomal miR-155 may serve as a bridge biomarker for cardio-reproductive risk. Inhibition of miR-155 in myocarditis models reduced macrophage infiltration and myocardial damage, suggesting a targetable node. However, given its broad immune role, off-target immunosuppression must be ruled out [49].

miR-146a functions as a negative feedback regulator of TLR/IL-1–NF-κB pathways by targeting IRAK1 and TRAF6. Genetic polymorphisms (e.g., rs2910164, rs2431697) influence miR-146a expression and correlate with cardiovascular risk in human populations [50]. Preclinical studies show that upregulation of miR-146a improves cardiac outcomes after ischemia–reperfusion injury. However, paradoxically, in pressure-overload heart failure models miR-146a overexpression impaired contractile function, highlighting the context-specific nature of its role [51].

The miR-34 family (including miR-34c) is intimately linked to the p53 pathway, cell cycle arrest, apoptosis and tissue aging. While miR-34a has been better studied in cardiac aging and stress, emerging evidence links miR-34c to reproductive organ aging and ovarian dysfunction [52]. Aberrant expression of miR-34c in endometrial/ovarian samples suggests potential as a biomarker for infertility due to premature ovarian insufficiency or endometriosis. Targeting the miR-34 family (mimics or inhibitors) may modulate both myocardial aging/remodeling and reproductive aging; however, data specific to miR-34c in the injured heart remain preliminary and hypothesis-forming [53].

## 7 miRNA biogenesis

Before delving into the specific roles of miRNAs in infertility and CVD, it is useful to briefly review how miRNAs are generated and processed in cells. Primary miRNA transcripts (pri-miRNAs) are usually transcribed by RNA polymerase II (Pol II) from either independent miRNA genes or from the introns of protein-coding genes (host genes) [31,32]. In some cases, RNA polymerase III can transcribe certain miRNAs, but Pol II is responsible for the majority of pri-miRNA synthesis. Pri-miRNAs are typically very long (often >1 kb) and contain a hairpin-loop structure. They undergo a two-step processing pathway, first in the nucleus, then in the cytoplasm.

In the nucleus, the enzyme Drosha (an RNase III endonuclease) and its cofactor DGCR8 (DiGeorge syndrome Critical Region 8, also known as Pasha) initiate miRNA processing. Drosha recognizes the characteristic hairpin within the pri-miRNA and cleaves near the base of the stem-loop, releasing a shorter ∼60–70 nt hairpin structure known as the precursor miRNA (pre-miRNA). This cleavage leaves the pre-miRNA with a 5′ phosphate and a 2-nucleotide 3′ overhang on its double-stranded stem. The remaining pri-miRNA flanking sequences are discarded. (An exception to this canonical pathway are mirtrons – miRNAs located in introns of host genes – which can bypass Drosha processing. Mirtrons are excised by the splicing machinery and debranched, directly forming a pre-miRNA-like hairpin [33,37].

**Fig. 2:**
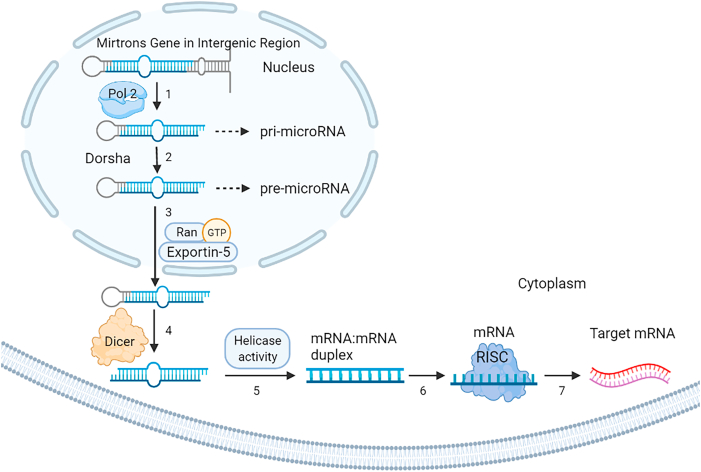
Biogenesis of miRNA. (1) RNA pol II is participated in transcription of pri-miRNA. (2) Drosha RNase III endonuclease cleaves the pri-miRNA close to the base of the primary stem-loop (3). The premiRNA is then transferred into the cytoplasm which becomes mature by RNase III endonuclease Dicer (4, 5). The loop region is identified by the helicase domain which further cleaves the two strands (6). The short-RNA fragment is recognized as miRNA which binds to the targeted mRNA in ordee to exert negative regulation on its expression (7). This figure adopted by Mohammadi et al. [32].

The pre-miRNA is then exported from the nucleus to the cytoplasm by the nuclear export protein Exportin-5 in a Ran-GTP–dependent manner [54]. Once in the cytoplasm, the enzyme Dicer (another RNase III endonuclease) binds to the pre-miRNA and performs the second cleavage. Dicer recognizes the 3′ overhang of the pre-miRNA and cuts approximately two helical turns (∼20 bp) away from the Drosha cleavage site, removing the terminal loop of the hairpin. This produces a short ∼22 nt RNA duplex consisting of two strands, the “guide” strand (mature miRNA) and the complementary “passenger” strand (often denoted miRNA*). The two strands of the duplex have the characteristics of miRNAs, the guide strand will be loaded into the RNA-induced silencing complex (RISC) to exert gene-silencing functions, whereas the passenger strand is usually degraded. (In some cases, the passenger strand can also have regulatory activity, but generally one strand is preferentially stabilized as the functional miRNA.) Dicer is a large multi-domain protein containing a helicase domain, a PAZ domain (which binds the 3′ overhang of RNA), two RNase III domains (which cut each strand of the duplex), and a double-stranded RNA-binding domain [55]. The PAZ domain anchors the pre-miRNA's 3′ end, and the distance to Dicer's RNase active sites determines where the cut is made, thus “measuring” the length of the miRNA duplex. The actions of Drosha and Dicer ensure a precise maturation of miRNAs from long transcripts to functional ∼22 nt RNA molecules. Interestingly, in plants the processing differs a Dicer homolog performs both steps in the nucleus, and a plant Exportin5 ortholog called HASTY exports the processed duplex to the cytosol [56,57]. The mature miRNA, once incorporated into RISC, guides the complex to target mRNAs by base-pairing with complementary sequences (often in the 3′ UTR of target genes), leading to translational repression or mRNA degradation. Fig. 2 summarizes the miRNA biogenesis pathway described above.

## 8 miRNAs in infertility and CVD

miRNAs have shown promise as novel biomarkers for both infertility and CVD, for use in diagnosis, prognosis, and even as therapeutic targets [25,58]. Several features make circulating miRNAs attractive candidates for biomarkers they are remarkably stable in plasma and serum, often exhibit tissue- or cell-specific expression patterns, resist degradation by RNases (especially when encapsulated in exosomes or bound to proteins), and can reflect ongoing pathological processes in real time. Moreover, changes in miRNA profiles can sometimes be detected earlier than changes in traditional protein biomarkers, suggesting they could serve as early warning signals for disease. For example, combining conventional biomarkers with miRNA signatures has been proposed as a way to improve long-term risk stratification in cardiovascular medicine. Likewise, in reproductive medicine, distinct miRNA expression patterns have been noted in conditions like PCOS and endometriosis, raising the possibility of using miRNAs to diagnose these causes of infertility [25].

Beyond their use as biomarkers, miRNAs themselves are being explored as therapeutic agents or targets. Because one miRNA can regulate multiple genes across different pathways, modulating a single miRNA can have broad effects on cellular function. This can be advantageous in complex disorders (like CVD or endometriosis) that involve dysregulation of entire gene networks. Researchers are developing miRNA-based therapeutics such as miRNA mimics (to restore the function of a downregulated miRNA) or miRNA inhibitors (antagomiRs to block an overactive miRNA). Early studies in models of heart disease and in fertility disorders have shown that altering specific miRNA levels can ameliorate disease features [58]. However, challenges remain in delivering these therapeutics specifically to target tissues and avoiding off-target effects. Novel computational tools and delivery platforms (including nanoparticles and engineered exosomes) are being investigated to enhance the precision of miRNA-based therapies.

## 9 Inflammatory miRNAs linking heart and reproductive health

Among the first miRNAs identified as a key player in cardiovascular inflammation was miR-155. miR-155 is known to be involved in immune cell differentiation and function, and it is upregulated during inflammatory responses [59,60]. Studies have implicated miR-155 in inflammatory heart conditions and linked it to reproductive hormone signaling, illustrating a possible connection between heart health and fertility. For example, Yan et al. (2016) reported significantly increased miR-155 levels in CD4^+^ T-cells infiltrating heart tissue in a mouse model of experimental autoimmune myocarditis. Similarly, Corsten et al. (2012) observed a strong up-regulation of miR-155 in acute viral myocarditis, in both susceptible mice and human patients. Importantly, inhibiting miR-155 in those mice reduced macrophage infiltration into the heart and led to less myocardial damage. These findings suggest that miR-155 contributes to cardiac inflammation and injury during myocarditis, and that targeting miR-155 can have cardioprotective effects [61,62].

A recent transcriptomic analysis of endomyocardial biopsy samples from patients with lymphocytic myocarditis identified a signature of 13 genes highly expressed during myocardial inflammation. Notably, seven of those genes (including SIGLEC1, TLR1, TLR2, TLR7, FCER1G, ITGB2, and CD14) are involved in innate immune activation, and their expression was linked to pathways that also induce miR-155. For instance, stimulation of toll-like receptors on dendritic cells and macrophages dramatically increases miR-155 expression [63]. Two of the genes from that signature, ITGB2 and SIGLEC1, encode surface molecules on antigen-presenting cells that trigger NF-κB–dependent inflammatory pathways the same pathways known to upregulate miR-155 synthesis [49]. Thus, miR-155 appears to be integrally connected to innate immune signaling networks in the heart.

miR-155 is also relevant to reproductive health. It is expressed in the testes and immune cells, and has been studied in the context of male fertility and conditions like PCOS in females. Elevated miR-155 levels have been observed in various LGSI conditions. As noted earlier, low testosterone levels in men are associated with subfertility and have been linked to chronic inflammation [64,65]. Interestingly, Murri et al. (2013) found a direct correlation between serum testosterone and miR-155 levels – PCOS patients had distinct miR-155 expression compared to healthy controls, with differences modulated by body mass index [66]. Additionally, Tsatsanis et al. (2015) reported that serum miR-155 could serve as a potential biomarker of male fertility, noting that low testosterone and increased inflammation often coincide with higher miR-155 in circulation [67]. These studies suggest that miR-155 may lie at the intersection of inflammatory cardiovascular pathology and reproductive endocrinology. In fact, a recent investigation directly examined miR-155's dual role in CVD and infertility, finding that this miRNA is a common factor in both pathologies (for example, through its effects on macrophage activation in atherosclerosis and on ovarian function in PCOS) [68]. Table 1 summarizes miR-155 and other selected miRNAs that have been implicated in both infertility and CVD.

**Table 1:** Various related miRNA s in collaboration infertility and cardiovascular disease.

miRNA	Expression	Model	Sample	Ref
miR-155-5p	Up	*Human*	Whole blood	[69]
miR-185-5p	Down	*Human*	Whole blood	[70]
miR-126/miR-145	Up	*Human*	Endometrial tissues	[71]
miR-200b/miR-204/miR-1827	Up	*Human*	Eutopic stromal and epithelial cells from endometriotic and normal patients.	[72]
miR-504	Down			
let-7c/miR-1296/miR-215	Aberrant methylation	*Human*	Cord blood	[66]

## 10 miR-185, miR-215 and female reproductive disorders

Another miRNA family of interest is the miR-185 family, which has been implicated in cardiac dysfunction as well as female reproductive disease. Using a cardiac-specific gene set analysis, Kim et al. (2015) identified miR-185 as an important regulator of cardiac hypertrophy; miR-185 targets multiple components of calcium signaling (such as the Na^+^/Ca^2+^ exchanger, CaMKIIδ, and NFAT) to exert an anti-hypertrophic effect [69]. In patients with dilated cardiomyopathy (DCM) a leading cause of heart failure, elevated miR-185 levels have been associated with better outcomes. For instance, Yu et al. (2016) found that patients with higher circulating miR-185 had significant improvements in left ventricular ejection fraction and decreases in heart failure biomarkers (like NT-proBNP) within one year, as well as reduced rates of hospitalization and cardiovascular death [73]. This suggests miR-185 may have a cardioprotective role. Conversely, in a myocardial infarction model, reduced miR-185 levels were linked to worsened cardiac repair loss of miR-185 led to increased expression of cathepsin K, which promoted angiogenesis but also potentially pathological remodeling; restoring miR-185 helped normalize this response [74,75]. Thus, maintaining appropriate miR-185 levels is important for cardiac homeostasis.

In the context of female infertility, miR-185 has been studied in endometriosis and PCOS. Endometriosis lesions are known to have aberrant angiogenesis and inflammation. Bioinformatic analyses have shown that VEGFA a key pro-angiogenic factor often overexpressed in endometriotic tissues – contains a conserved binding site for miR-185 in its 3′ untranslated region [76,77]. Consistently, Razi et al. (2019) reported that miR-185-5p expression was downregulated in the blood and endometrial samples of women with endometriosis [78]. The downregulation of miR-185 could lead to unchecked expression of VEGFA and other target genes, contributing to the excessive angiogenesis and lesion growth seen in endometriosis. In line with this, miR-185 is thought to act as a brake on pathological angiogenesis.

miR-185 may also have a protective role in polycystic ovarian syndrome (PCOS). Prior studies in metabolic disease models have shown that miR-185 can improve insulin sensitivity and reduce liver steatosis. Chen et al. (2019) found that increasing miR-185 in insulin-resistant liver cells improved their insulin response, speculating that miR-185 might similarly benefit metabolic disturbances in PCOS. In PCOS, chronic low-grade inflammation and angiogenic imbalance in ovarian tissue are common. One study noted that miR-185 overexpression could reduce the production of VEGFA (likely by targeting the VEGFA mRNA directly), which in turn could normalize the angiogenic environment in the ovaries [79,80]. Since VEGFA is crucial for blood vessel formation and ovarian function, an excess can exacerbate PCOS features. By targeting VEGFA and possibly components of the PI3K/AKT signaling pathway, miR-185 might prevent the abnormal follicular angiogenesis and ovarian dysfunction in PCOS. Indeed, experimental overexpression of VEGFA was shown to reverse the inhibitory effects of miR-185 on endothelial cell proliferation and migration, reinforcing that miR-185's effects are at least partly mediated through VEGFA. Additionally, miR-185 has been reported to target PI3K (phosphoinositide-3-kinase), thereby influencing autophagy and oxidative stress pathways in cardiomyocytes [79]; such pathways are also relevant in ovarian aging and PCOS, suggesting further avenues by which miR-185 links cardiac and ovarian health.

Another miRNA that links infertility and inflammation is miR-215-5p. miR-215-5p has known roles in cancer biology, but recent work implicates it in endometriosis and possibly early embryonic development. Abnormally low expression of miR-215-5p has been observed in endometriotic tissues [81]. Wu et al. (2022) found that miR-215-5p was reduced in serum exosomes of endometriosis patients, correlating with disease severity [81]. One of the major targets of miR-215-5p is CXCL2 (also known as MIP-2α), a chemokine involved in neutrophil recruitment and angiogenesis [70,82]. Overexpression of CXCL2 has been detected in the peritoneal fluid of women with endometriosis, contributing to the pro-inflammatory peritoneal environment [80]. When miR-215-5p is silenced or downregulated (as in endometriosis), CXCL2 is overexpressed, which can facilitate the progression of endometriotic lesions by enhancing inflammation and neovascularization [81]. Interestingly, aberrant miR-215 levels might also have consequences for offspring differential miR-215 expression has been linked to altered DNA methylation patterns in children conceived via intracytoplasmic sperm injection (ICSI), although the exact implications of this are still being investigated [83]. Overall, miR-215-5p serves as another example of a miRNA that can influence both reproductive pathology (endometriosis, infertility) and processes relevant to cardiovascular biology (inflammation and angiogenesis).

## 11 Exosomal miRNAs in infertility and CVD

**Fig. 3:**
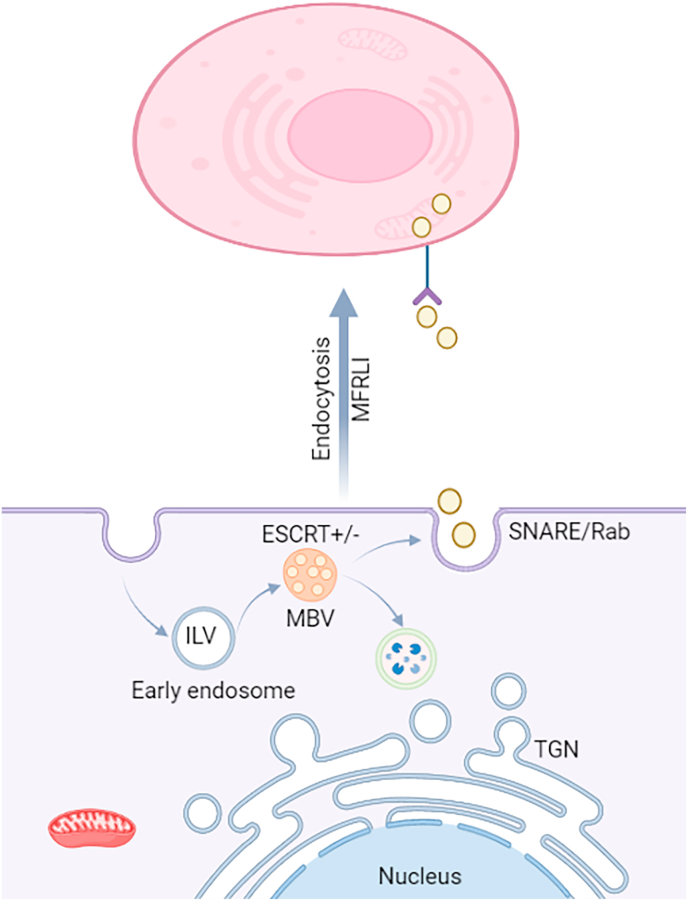
Cargo loading, biogenesis, and secretion of exosomes. intraluminal vesicles)ILVs) are created by invagination of endocytic membrane that subsequently leads to the exosome secretion. Cargo molecules including lipids, RNAs and proteins are loaded into ILVs through an ESCRT-dependent or -independent pathway during maturation process. Maturation of initial endosomes leads to the formation of multi-vesicular bodies (MVBs) which are transferred to the trans-Golgi network (TGN) to recycle the endosomes. Degradation could be occurred following delivery of the MVBs to lysosome. The MVBs can be moved along micro-tubules to release the exosome into extra-cellular compartment following fusion with plasma membrane. Furthermore, fusion of MVBs with plasma membrane involves the SNARE and Rab GTPase complexes. Ultimately, exosomal contents originated from source cells might be taken up by target cells through endocytosis, receptor-ligand interactions (MFRLI) or direct membrane fusion. This figure adopted by Balandeh et al. [11].

Exosomes are extracellular vesicles (∼40–150 nm in diameter) that play a key role in intercellular communication by transporting proteins, lipids, and nucleic acids (including miRNAs) between cells. Given that miRNAs are often packaged into exosomes, understanding exosome biology is important for appreciating how miRNA signals might be exchanged between the reproductive and cardiovascular systems. Exosomes originate from the endosomal pathway, they form inside cells when early endosomes mature into multivesicular bodies (MVBs) containing intraluminal vesicles (ILVs). When an MVB fuses with the plasma membrane, it releases those ILVs as exosomes into the extracellular space (Fig. 3). The formation and release of exosomes involve a complex machinery called ESCRT (Endosomal Sorting Complex Required for Transport), which has four protein complexes (0, I, II, III) that help cluster cargo and pinch off vesicles [[84], [85], [86]]. Briefly, ESCRT-0 recognizes and sequesters ubiquitinated cargo on the endosome membrane; ESCRT-I and II then facilitate the inward budding of the endosomal membrane, forming ILVs; finally, ESCRT-III (with the ATPase VPS4) drives membrane scission, cutting off the ILVs, and is then recycled [87]. Notably, cells can also load cargo (like miRNAs) into exosomes via ESCRT-independent mechanisms involving specific lipids or RNA-binding proteins, but in either case, certain miRNAs are selectively packaged while others are not a process that is the subject of ongoing research.

Once secreted, exosomes can travel through bodily fluids and be taken up by recipient cells, where they unload their cargo and thereby modulate the function of the target cell. In the context of infertility and CVD, exosomal miRNAs could conceivably mediate cross-talk between reproductive organs and the cardiovascular system. For example, an inflammatory state in the reproductive tract (such as endometriosis) could release exosomes carrying miRNAs that affect distant vascular or cardiac cells, or vice versa, a cardiovascular condition could influence ovarian function via circulating exosomes. While this systemic exchange is still hypothetical, there is direct evidence of exosomal miRNAs playing roles within each condition.

One extensively studied miRNA in exosomes is miR-21. The gene for miR-21 (on chromosome 17q23.2) is highly conserved and miR-21 is abundantly expressed in multiple cardiovascular cell types (vascular smooth muscle cells, endothelial cells, cardiomyocytes, and especially cardiac fibroblasts) [45,88,89]. miR-21 is known as a pro-fibrotic and pro-angiogenic miRNA in the heart. Transgenic mice overexpressing miR-21 show reduced fibrosis and smaller infarct sizes after myocardial infarction, due in part to miR-21's ability to inhibit pro-apoptotic and pro-fibrotic targets (e.g., FasL, PTEN, Sprouty) and thereby enhance cell survival and angiogenesis [90]. In contrast, pharmacological inhibition of miR-21 has been found to attenuate cardiac fibrosis in some studies, indicating its complex role in cardiac remodeling.

Importantly, miR-21 has also been implicated in female reproductive disorders. Harp et al. (2016) discovered that exosomes derived from endometrial stromal cells of endometriosis patients have an altered miRNA cargo profile compared to exosomes from women without endometriosis. Among the differentially expressed miRNAs, miR-21 was significantly upregulated in exosomes from endometriosis patients [91]. Since miR-21 promotes angiogenesis, its higher presence in endometriosis-derived exosomes may contribute to the abnormal blood vessel growth seen in endometriotic lesions. These exosomes can be found in the peritoneal fluid and uterine microenvironment, where they may be taken up by nearby cells (such as endothelial cells or immune cells), potentially exacerbating the pathological processes of endometriosis – for example, by enhancing angiogenesis or inflammation that supports lesion growth and implantation failure. Thus, exosomal miR-21 represents a mechanistic link between a reproductive disorder (endometriosis-related infertility) and processes (angiogenesis, fibrosis) that are also critical in cardiovascular disease. Table 2 highlights miR-21 and other exosomal miRNAs that have been associated with infertility and CVD. While early post-injury upregulation in cardiac fibroblasts can limit apoptosis and foster angiogenesis (PTEN/FasL/Sprouty pathways), sustained activation favors extracellular matrix deposition and fibrosis. This dualism explains apparently contradictory reports and underscores the need for stage and cell-specific targeting.

**Table 2:** Various related exosomal miRNA s in collaboration infertility and cardiovascular disease.

Cargo	Expression	Model	Cell line	Ref
miR-21	Up	*In vivo (Human)*	ESCs and HUVECs	[92]
miR-126	No different			
Responsive-miR-451	Up	*In vivo (Mouse)*	CPC	[93]
miR-126	Up	*In vivo (Human and Mice)*	PBMC	[94]

Human aortic smooth muscle cells (HASMCs), Human cardiac microvascular endothelial cell (HCMECs), Human primary endometrial stromal cells (ESCs), human umbilical vein endothelial cells (HUVECs), Cardiac progenitors (CPC), peripheral blood mononuclear cell (PBMC).

Another intriguing connection involves the transcription factor Krüppel-like factor 5 (KLF5) and exosomal miR-155. KLF5 is a regulator of cell proliferation, differentiation, and inflammation [95]. It has important roles in both implantation and cardiovascular pathology. In the uterus, KLF5 is required for proper implantation and decidualization. Conditional deletion of Klf5 in the mouse uterus led to implantation failure due to poor uterine receptivity and deficient decidual response. KLF5 regulates the expression of COX-2 (an enzyme crucial for prostaglandin production during implantation); without KLF5, COX-2 levels in the uterine epithelium drop, leading to implantation defects [92,[96], [97], [98], [99], [100], [101], [102], [103]]. KLF5 also influences the transformation of endometrial stromal cells during decidualization, and its absence results in fewer decidual cells and increased pregnancy loss [97,98]. These findings underscore KLF5's role in fertility. Meanwhile, in the cardiovascular system, KLF5 is activated by stress stimuli such as angiotensin II and oxidative stress and contributes to vascular remodeling. It is upregulated in vascular smooth muscle cells (VSMCs) during atherosclerosis and after vascular injury. Oxidized low-density lipoprotein (a key factor in atherogenesis) induces KLF5 in VSMCs, which can worsen endothelial dysfunction [93].

Notably, KLF5 appears to control the production and release of miR-155-rich exosomes from VSMCs. Overexpression of KLF5 in VSMCs led to enhanced transcription of miR-155, and these cells secreted exosomes enriched in miR-155. When endothelial cells were exposed to exosomes from KLF5-overexpressing VSMCs, they exhibited reduced proliferation, increased permeability, and decreased tight junction proteins changes that mirror endothelial dysfunction in atherosclerosis [92]. In essence, KLF5 creates a paracrine loop, atherosclerotic stimuli activate KLF5 in VSMCs; KLF5 induces miR-155 expression and packaging into exosomes; those exosomes then impair neighboring endothelial cells via miR-155. This mechanism is not only relevant to vascular disease but also ties back to fertility since, as discussed, miR-155 and inflammatory pathways play roles in conditions like PCOS and male infertility. Therefore, factors like KLF5 and miR-155 exemplify how a molecular pathway can have dual relevance contributing to atherosclerosis in the vasculature and to inflammation-related infertility in the reproductive system.

## 12 Therapeutic applications of miRNAs and exosomal miRNAs in CVD and infertility

The recognition that miRNAs and exosomal miRNAs contribute to the pathophysiology of both CVD and infertility has spurred interest in harnessing them for therapy. One approach is cell-based or exosome-based therapy. For instance, researchers have explored using exosomes derived from stem cells or progenitor cells as natural delivery vehicles to repair damaged heart tissue or improve reproductive outcomes. Cardiac progenitor cell (CPC)-derived exosomes have shown cardioprotective effects. CPC exosomes can be readily taken up by cardiomyocytes and have been reported to protect these heart cells from oxidative stress in vitro and to reduce injury from ischemia–reperfusion in vivo [102]. Remarkably, CPC exosomes are enriched in miR-144/miR-451 (particularly miR-451a) – a cluster of miRNAs regulated by the cardiac transcription factor GATA4 [101,102]. miR-451 has been demonstrated to protect cardiomyocytes against simulated ischemia/reperfusion-induced cell death [92]. Interestingly, CPC exosomes contain abundant miR-451 but very little miR-144, suggesting selective packaging. By delivering miR-451 and other cardioprotective molecules, CPC-derived exosomes can ameliorate myocardial ischemic injury – akin to the benefits seen with CPC transplantation itself (which is known to reduce apoptosis and improve cardiac function in infarct models) [103]. Because exosomes are small lipid vesicles, they can circulate and penetrate tissues efficiently, making them attractive vehicles for therapeutic delivery of RNAs. The exact mechanisms by which specific miRNAs are sorted into exosomes remain an area of active research. Unraveling these mechanisms could allow scientists to engineer exosomes with custom miRNA cargoes optimized to treat particular conditions (for example, loading exosomes with a miR-155 inhibitor to target inflammation in both heart and reproductive tissues) [93].

On the infertility side, there is also exploration of miRNA-based interventions. For example, in endometriosis, where aberrant miRNAs like miR-21 and miR-451a are involved, researchers are testing whether antagomiRs (antisense oligonucleotides that specifically inhibit miRNAs) against those miRNAs can reduce lesion growth or alleviate symptoms. Likewise, for PCOS, strategies to boost protective miRNAs (such as miR-185 or let-7 family members) or suppress detrimental ones (such as miR-155 or others involved in insulin signaling) are being considered. In male infertility related to metabolic disease (e.g. diabetes-induced testicular damage), studies have identified miR-504 and miR-935 as potential therapeutic targets, inhibiting these miRNAs in diabetic mouse models helped reverse testicular cell apoptosis and improved sperm parameters. This underscores the potential of miRNA modulators as therapy.

It is worth noting that miRNA-based therapies for humans are still largely in experimental or clinical trial phases. One challenge is ensuring specific delivery to the intended tissue (heart or reproductive organs) to avoid off-target effects, since miRNAs can have different roles in different cell types. Using exosomes as delivery vehicles is a promising solution to this, as exosomes can be engineered to display surface molecules that direct them to certain cell types (for instance, an exosome could be modified to home to inflamed endometrium or to injured myocardium). Additionally, exosomes naturally protect RNA cargo from degradation and are generally well-tolerated by the immune system. Clinical trials are already underway testing exosomes or exosome-mimetics loaded with therapeutic cargo for heart disease, and similar approaches could be envisioned for treating infertility (e.g., intrauterine infusion of exosomes to improve endometrial receptivity).

In summary, leveraging miRNAs and exosomal miRNAs for therapy represents a cutting-edge interdisciplinary approach. By targeting common molecular denominators of infertility and CVD, such therapies have the potential to simultaneously improve reproductive outcomes and cardiovascular health in affected individuals – literally addressing the “injured heart” of an infertile patient at both a symbolic and molecular level. Continued research is needed to translate these findings into safe and effective treatments.

## 13 Conclusions

MiRNAs, as a class of non-coding RNAs, have emerged as pivotal regulators of gene expression post-transcription. They modulate numerous fundamental physiological processes – including apoptosis, differentiation, and proliferation and are involved in diverse pathological processes such as inflammation, angiogenesis, and oncogenesis. Accumulating evidence indicates that miRNAs (and other non-coding RNAs) directly contribute to key pathways in both CVD and infertility. For example, miRNAs influence hormonal signaling, vascular function, and inflammatory pathways that are common to heart disease and reproductive disorders. Discovering the underlying mechanisms by which specific miRNAs operate in these intertwined processes is of great importance, as it opens the door to novel therapeutic strategies and preventive measures for patients.

Within the human genome, many non-coding RNAs have roles in the development of CVD and infertility, although the exact mechanisms of action for most are still being elucidated. One intriguing hypothesis is that some of these RNAs act as messengers between organ systems. For instance, miRNAs may be secreted in exosomes by one tissue (such as the cardiovascular system) and taken up by another (such as the ovaries or endometrium), thereby exerting effects beyond their cell of origin. Exosomes, due to their natural role in intercellular communication, could be harnessed to deliver therapeutic RNAs. In the future, we may be able to use exosome-based delivery of specific miRNA mimics or inhibitors as a means to combat infertility and CVD simultaneously for example, an exosome carrying cardioprotective and pro-fertility miRNAs to a patient who has both conditions.

In conclusion, miRNAs serve as a molecular link between the injured heart and the infertile reproductive system. Understanding this link provides a unique perspective that could revolutionize how we approach women's health, emphasizing the need to view cardiovascular and reproductive health in an integrated manner. Ongoing research into miRNAs and exosomal communication holds promise for identifying early biomarkers of disease risk (with infertility potentially flagging future CVD, or vice versa) and for developing innovative treatments that improve the overall health and quality of life of patients facing these challenging conditions.

## Ethics approval and consent to participate

Not applicable.

## Consent for publication

Not applicable.

## Availability of data and materials

Not applicable.

## Funding

Not applicable.

## CRediT authorship contribution statement

**Amir Hossein Mohammadi:** Conceptualization, Data curation, Investigation, Methodology, Project administration, Software, Supervision, Validation, Visualization, Writing – original draft, Writing – review & editing. **Parvin Yavari:** Writing – original draft.

## Declaration of competing interest

The authors had no competing interests.

## Data Availability

No data was used for the research described in the article.
